# Supplementary dataset for child and adult exposure and health risk evaluation following the use of metal- and metalloid-containing costume cosmetics sold in the United States

**DOI:** 10.1016/j.dib.2017.04.033

**Published:** 2017-05-04

**Authors:** Angela L. Perez, Melanie Nembhard, Andrew Monnot, Daniel Bator, Elizabeth Madonick, Shannon H. Gaffney

**Affiliations:** a Cardno ChemRisk, LLC, 101 2nd Street, Suite 700, San Francisco, CA 94105, United States; b Dept. of Environmental Health Sciences, University of Michigan School of Public Health, 1415 Washington Heights, Ann Arbor, MI 48109-2029, United States; c Brooks Applied Labs, 3958 6th Ave NW, Seattle, WA 98107, United States

**Keywords:** Cosmetics, Makeup, Halloween, Metal, Metalloid, Lead, Arsenic, Face paint, Body paint

## Abstract

The data presented in this article are related to the research article entitled "Child and adult exposure and health risk evaluation following the use of metal- and metalloid-containing costume cosmetics sold in the United States" [1]. This article describes the concentration of metals and metalloids contained in various cosmetic products such as body paint, lipstick and eye shadow, the relative percent deviation of two analyses performed on the products and the physico-chemico properties of the metals and metalloids used in the SkinPerm model presented in the aforementioned article.

**Specifications Table**

**Table:** 

**Subject area**	Exposure, Health Risk Evaluation
**More specific subject area**	Metals in Costume Cosmetics
**Type of data**	Tables, Text file
**How data was acquired**	X-ray Fluorescence was used as an initial measurement of concentration of the metals;
	Inductively-Coupled Plasma Mass Spectrometry;
	Inductively-Coupled Plasma Triple Quadrupole Mass Spectrometry;
	Research
**Data format**	Raw
**Experimental factors**	Digestion of the products was used to determine the concentrations of the metals and metalloids in the various metals
**Experimental features**	The concentrations of different metals and metalloids in various costume cosmetics was determined for a child and adult intermittent user and an adult occupational user of the products
**Data source location**	San Francisco, CA
**Data accessibility**	The data are available with this article

**Value of the data**

• Provides an exposure assessment on realistic exposure scenarios and for multiple age use categories
• Risk assessment based on multiple routes of exposure, including dermal uptake and hand to mouth which have previously not been published
• Intra-lab comparison using two similar methodologies to evaluate how the sensitivity of the method impacts the ability to calculate an assessed risk
• Data from creams, sticks and lip products can be compared against other existing data for similar cosmetic products to estimate cumulative exposures
• Serves as a benchmark for other researchers to evaluate the exposure potential and risk for similar cosmetic products with chemicals not tested in our assessment, including organic chemicals

## 1 Data

The dataset of this article provides the concentration of the metals and metalloids in the various products from the three different methods used to measure concentration (Tables S1, S2 and S3), along with the relative percent deviation of concentration measurements between the inductively-coupled plasma mass spectrometry and the inductively-coupled plasma triple quadrupole mass spectrometry analyses (Table S4) and the physico-chemico properties of each metal/metalloid used in the SkinPerm modeling (Table S5).

## 2 Experimental design, materials and methods

Each cosmetic sample was pre-screened for metal content with the use of an X-ray fluorescence analyzer. If there was any metal detected in a cosmetic then it was purchased in order to do further analysis. Each cosmetic sample purchased was analyzed a second time with the X-ray fluorescence analyzer in a controlled environment and the data is shown in Table S1. Twenty-six samples from those analyzed by X-ray fluorescence were randomly selected and sent to EMSL, Analytical Inc. (Cinnaminson, NJ, USA) where they were analyzed by inductively-coupled plasma mass spectrometry. A subset of ten samples, five eyeshadows and five body paints, were selected from those sent to EMSL Laboratory, and they were sent to Brooks Applied Labs, for additional testing by inductively-coupled plasma triple quadrupole mass spectrometry. The additional testing included analysis for 67 metals and metalloids, including Hg and methyl mercury (MeHg). The data from the inductively-coupled plasma mass spectrometry and inductively-coupled plasma triple quadrupole mass spectrometry, for the ten samples analyzed by both methods, are shown in Table S2 and Table S3, respectively. The relative percent deviation between the data from EMSL and Brooks Applied Labs are presented in Table S4. Finally, SkinPerm modeling was performed for each chemical to determine the average systemic absorption dose of each chemical. To perform the SkinPerm model, the physico-chemico parameters for each element were needed. These parameters are presented in Table S5. The SkinPerm model results can be found in [1].
