# Supplementary material for: Supplementary dataset for child and adult exposure and health risk evaluation following the use of metal- and metalloid-containing costume cosmetics sold in the United States
Source: Data Brief. 2017 May 4;13:129–31. doi: 10.1016/j.dib.2017.04.033 (PMC5451178; doi:10.1016/j.dib.2017.04.033)
Supplement: Supplementary file 2 — Supplementary material [file mmc2.docx]

Table S1. Metal and metalloid concentrations in costume cosmetics as measured using X-ray fluorescence (mg/kg)

|  | | | | | | | | | | | | | | | | | | | |
| --- | --- | --- | --- | --- | --- | --- | --- | --- | --- | --- | --- | --- | --- | --- | --- | --- | --- | --- | --- |
| **Cosmetic Product Category*** | **Country of Origin** | **Pb** | **Pb +/-** | **Hg** | **Hg +/-** | **As** | **As +/-** | **Cd** | **Cd +/-** | **Ni** | **Ni +/-** | **Co** | **Co +/-** | **Cr** | **Cr +/-** | **Sb** | **Sb +/-** | **Ag** | **Ag +/-** |
| Body paint | USA | <LOD | 6 | <LOD | 4 | <LOD | 2 | <LOD | 65 | <LOD | 44 | <LOD | 36 | <LOD | 51 | <LOD | 147 | 208 | 21 |
| Body paint | USA | <LOD | 7 | <LOD | 7 | <LOD | 3 | <LOD | 79 | <LOD | 54 | <LOD | 53 | <LOD | 56 | <LOD | 177 | 249 | 25 |
| Body paint | USA | 29 | 5 | <LOD | 47 | <LOD | 8 | <LOD | 152 | <LOD | 285 | <LOD | 363 | <LOD | 331 | <LOD | 374 | 278 | 47 |
| Body paint | USA | <LOD | 7 | <LOD | 5 | 3 | 1 | <LOD | 75 | <LOD | 53 | <LOD | 54 | <LOD | 64 | <LOD | 175 | 331 | 24 |
| Body paint | USA | <LOD | 5 | <LOD | 4 | 2 | 1 | <LOD | 60 | 67 | 15 | <LOD | 25 | 64 | 20 | <LOD | 137 | 274 | 19 |
| Body paint | USA | <LOD | 6 | <LOD | 5 | <LOD | 2 | <LOD | 77 | <LOD | 51 | <LOD | 31 | <LOD | 89 | <LOD | 182 | 328 | 24 |
| Body paint | USA | <LOD | 5 | <LOD | 4 | <LOD | 2 | <LOD | 55 | <LOD | 38 | <LOD | 24 | <LOD | 31 | <LOD | 117 | 88 | 18 |
| Body paint | USA | <LOD | 5 | <LOD | 4 | <LOD | 2 | <LOD | 48 | <LOD | 31 | <LOD | 20 | <LOD | 31 | <LOD | 102 | 57 | 16 |
| Body paint | USA | <LOD | 4 | <LOD | 2 | <LOD | 1 | <LOD | 38 | <LOD | 25 | <LOD | 15 | <LOD | 24 | <LOD | 82 | 53 | 12 |
| Body paint | Taiwan | <LOD | 12 | <LOD | 11 | <LOD | 5 | <LOD | 125 | <LOD | 107 | <LOD | 260 | <LOD | 163 | <LOD | 299 | 379 | 39 |
| Body paint | USA | 27 | 4 | <LOD | 36 | <LOD | 6 | <LOD | 114 | <LOD | 196 | <LOD | 253 | <LOD | 221 | <LOD | 276 | 257 | 36 |
| Body paint | USA | <LOD | 6 | <LOD | 5 | 2 | 1 | <LOD | 71 | <LOD | 48 | <LOD | 34 | <LOD | 70 | <LOD | 164 | 291 | 22 |
| Body paint | USA | <LOD | 6 | <LOD | 4 | <LOD | 2 | <LOD | 59 | <LOD | 40 | <LOD | 21 | <LOD | 37 | <LOD | 130 | 128 | 19 |
| Body paint | USA | <LOD | 6 | <LOD | 4 | <LOD | 2 | <LOD | 64 | <LOD | 45 | <LOD | 30 | <LOD | 42 | <LOD | 142 | 131 | 21 |
| Body paint | China | <LOD | 6 | <LOD | 6 | <LOD | 2 | <LOD | 79 | <LOD | 89 | <LOD | 475 | <LOD | 117 | <LOD | 184 | 359 | 25 |
| Body paint | China | <LOD | 4 | <LOD | 3 | 2 | 1 | <LOD | 57 | <LOD | 39 | <LOD | 47 | <LOD | 46 | <LOD | 123 | 151 | 18 |
| Body paint | China | <LOD | 5 | <LOD | 5 | 2 | 1 | <LOD | 64 | <LOD | 45 | <LOD | 40 | <LOD | 47 | <LOD | 144 | 250 | 20 |
| Body paint | USA | <LOD | 7 | <LOD | 6 | <LOD | 3 | <LOD | 79 | 91 | 28 | <LOD | 403 | <LOD | 103 | <LOD | 176 | 204 | 25 |
| Body paint | USA | <LOD | 8 | <LOD | 6 | <LOD | 3 | <LOD | 81 | <LOD | 57 | <LOD | 34 | <LOD | 63 | <LOD | 179 | 213 | 26 |
| Body paint | USA | <LOD | 5 | <LOD | 4 | <LOD | 2 | <LOD | 58 | <LOD | 38 | <LOD | 26 | 15198 | 212 | <LOD | 128 | 147 | 19 |
| Body paint | USA | <LOD | 5 | <LOD | 4 | <LOD | 2 | <LOD | 54 | <LOD | 36 | <LOD | 22 | <LOD | 36 | <LOD | 117 | 164 | 17 |
| Body paint | USA | <LOD | 6 | <LOD | 4 | <LOD | 2 | <LOD | 64 | <LOD | 41 | <LOD | 27 | <LOD | 35 | <LOD | 138 | 99 | 20 |
| Body paint | USA | <LOD | 5 | <LOD | 3 | <LOD | 2 | <LOD | 51 | <LOD | 36 | <LOD | 46 | <LOD | 54 | <LOD | 113 | 92 | 17 |
| Body paint | USA | <LOD | 5 | <LOD | 3 | 3 | 1 | <LOD | 58 | <LOD | 40 | <LOD | 31 | <LOD | 53 | <LOD | 131 | 181 | 18 |
| Body paint | USA | <LOD | 5 | <LOD | 4 | <LOD | 2 | <LOD | 54 | <LOD | 37 | <LOD | 57 | <LOD | 37 | <LOD | 119 | 123 | 17 |
| Body paint | USA | <LOD | 7 | <LOD | 4 | <LOD | 2 | <LOD | 72 | <LOD | 54 | <LOD | 171 | 23668 | 356 | <LOD | 161 | 251 | 23 |
| Body paint | USA | <LOD | 6 | <LOD | 5 | <LOD | 2 | <LOD | 67 | <LOD | 53 | <LOD | 160 | <LOD | 62 | <LOD | 150 | 223 | 21 |
| Body paint | China | <LOD | 6 | <LOD | 5 | <LOD | 2 | <LOD | 71 | <LOD | 50 | <LOD | 49 | <LOD | 56 | <LOD | 157 | 182 | 23 |
| Body paint | China | <LOD | 8 | <LOD | 5 | <LOD | 3 | <LOD | 86 | 100 | 29 | <LOD | 435 | <LOD | 116 | <LOD | 192 | 188 | 28 |
| Body paint | China | <LOD | 7 | <LOD | 5 | <LOD | 2 | <LOD | 70 | <LOD | 52 | <LOD | 99 | <LOD | 55 | <LOD | 153 | 130 | 23 |
| Body paint | USA | <LOD | 8 | <LOD | 6 | <LOD | 3 | <LOD | 90 | <LOD | 113 | <LOD | 645 | <LOD | 144 | <LOD | 210 | 344 | 28 |
| Body paint | USA | <LOD | 5 | <LOD | 4 | <LOD | 2 | <LOD | 58 | <LOD | 39 | <LOD | 23 | <LOD | 38 | <LOD | 127 | 194 | 18 |
| Body paint | USA | <LOD | 5 | <LOD | 4 | <LOD | 2 | <LOD | 65 | <LOD | 42 | <LOD | 26 | <LOD | 40 | <LOD | 143 | 182 | 21 |
| Body paint | USA | <LOD | 5 | <LOD | 4 | <LOD | 2 | <LOD | 63 | <LOD | 43 | <LOD | 27 | <LOD | 40 | <LOD | 140 | 220 | 20 |
| Body paint | USA | <LOD | 5 | <LOD | 3 | <LOD | 2 | <LOD | 54 | <LOD | 34 | <LOD | 19 | <LOD | 33 | <LOD | 119 | 171 | 17 |
| Body paint | USA | <LOD | 6 | <LOD | 5 | <LOD | 2 | <LOD | 66 | 48 | 16 | <LOD | 39 | <LOD | 47 | <LOD | 148 | 211 | 21 |
| Body paint | USA | <LOD | 5 | <LOD | 4 | <LOD | 2 | <LOD | 60 | <LOD | 41 | <LOD | 72 | <LOD | 40 | <LOD | 132 | 193 | 19 |
| Body paint | USA | <LOD | 6 | <LOD | 4 | <LOD | 2 | <LOD | 68 | <LOD | 47 | <LOD | 29 | <LOD | 41 | <LOD | 150 | 161 | 22 |
| Body paint | USA | <LOD | 4 | <LOD | 4 | 2 | 1 | <LOD | 57 | <LOD | 39 | <LOD | 21 | <LOD | 40 | <LOD | 126 | 171 | 18 |
| Body paint | USA | <LOD | 6 | <LOD | 5 | <LOD | 2 | <LOD | 81 | <LOD | 61 | <LOD | 163 | 25697 | 417 | <LOD | 188 | 304 | 25 |
| Body paint | USA | <LOD | 8 | <LOD | 7 | <LOD | 3 | <LOD | 94 | <LOD | 68 | <LOD | 117 | <LOD | 64 | <LOD | 212 | 228 | 30 |
| Body paint | USA | <LOD | 6 | <LOD | 5 | <LOD | 2 | <LOD | 69 | <LOD | 46 | <LOD | 30 | <LOD | 43 | <LOD | 155 | 210 | 22 |
| Body paint | USA | <LOD | 7 | <LOD | 6 | <LOD | 2 | <LOD | 83 | <LOD | 57 | <LOD | 38 | <LOD | 65 | <LOD | 189 | 279 | 27 |
| Body paint | USA | <LOD | 6 | <LOD | 5 | <LOD | 2 | <LOD | 79 | <LOD | 85 | <LOD | 448 | <LOD | 116 | <LOD | 187 | 313 | 25 |
| Body paint | USA | <LOD | 6 | <LOD | 4 | <LOD | 2 | <LOD | 65 | <LOD | 41 | 34 | 11 | <LOD | 50 | <LOD | 146 | 183 | 21 |
| Body paint | USA | <LOD | 7 | <LOD | 6 | <LOD | 3 | <LOD | 82 | 94 | 23 | <LOD | 160 | <LOD | 91 | <LOD | 197 | 318 | 26 |
| Body paint | USA | <LOD | 7 | 7 | 2 | 3 | 1 | <LOD | 77 | 65 | 19 | <LOD | 110 | <LOD | 79 | <LOD | 181 | 390 | 24 |
| Body paint | USA | <LOD | 9 | <LOD | 8 | <LOD | 3 | <LOD | 95 | 116 | 36 | <LOD | 524 | <LOD | 141 | <LOD | 232 | 411 | 30 |
| Body paint | USA | <LOD | 10 | <LOD | 10 | <LOD | 4 | <LOD | 97 | 151 | 30 | <LOD | 52 | 421 | 94 | <LOD | 240 | 478 | 34 |
| Body paint | USA | <LOD | 10 | <LOD | 8 | <LOD | 4 | <LOD | 92 | <LOD | 74 | <LOD | 60 | <LOD | 116 | <LOD | 234 | 413 | 33 |
| Body paint | USA | <LOD | 8 | <LOD | 8 | <LOD | 3 | <LOD | 79 | 114 | 24 | <LOD | 69 | <LOD | 88 | <LOD | 198 | 389 | 28 |
| Body paint | USA | <LOD | 16 | <LOD | 17 | <LOD | 7 | <LOD | 89 | <LOD | 100 | <LOD | 81 | <LOD | 454 | <LOD | 251 | 341 | 42 |
| Body paint | USA | <LOD | 16 | <LOD | 18 | <LOD | 7 | <LOD | 87 | <LOD | 103 | <LOD | 198 | <LOD | 357 | <LOD | 243 | 395 | 40 |
| Body paint | USA | <LOD | 17 | <LOD | 20 | <LOD | 7 | <LOD | 83 | <LOD | 121 | <LOD | 516 | <LOD | 452 | <LOD | 233 | 326 | 40 |
| Body paint | USA | <LOD | 6 | <LOD | 5 | <LOD | 2 | <LOD | 66 | <LOD | 50 | <LOD | 41 | <LOD | 50 | <LOD | 155 | 277 | 22 |
| Eye shadow | China | <LOD | 10 | <LOD | 8 | <LOD | 3 | <LOD | 67 | 68 | 18 | <LOD | 97 | <LOD | 55 | <LOD | 160 | 409 | 21 |
| Eye shadow | China | <LOD | 7 | 9 | 2 | <LOD | 3 | <LOD | 70 | 75 | 18 | <LOD | 97 | <LOD | 59 | <LOD | 167 | 386 | 22 |
| Eye shadow | China | <LOD | 6 | 6 | 2 | <LOD | 2 | <LOD | 66 | <LOD | 47 | <LOD | 93 | <LOD | 51 | <LOD | 157 | 369 | 21 |
| Eye shadow | China | <LOD | 6 | <LOD | 5 | 3 | 1 | <LOD | 68 | 74 | 17 | <LOD | 75 | <LOD | 50 | <LOD | 160 | 394 | 21 |
| Eye shadow | China | <LOD | 6 | 7 | 2 | 4 | 1 | <LOD | 72 | 59 | 17 | <LOD | 99 | <LOD | 53 | <LOD | 169 | 389 | 22 |
| Eye shadow | China | <LOD | 6 | 8 | 2 | <LOD | 2 | <LOD | 68 | 71 | 17 | <LOD | 80 | <LOD | 48 | <LOD | 159 | 395 | 21 |
| Eye shadow | China | 18 | 6 | <LOD | 10 | <LOD | 6 | <LOD | 122 | <LOD | 214 | <LOD | 1344 | <LOD | 287 | <LOD | 308 | 480 | 38 |
| Eye shadow | China | <LOD | 7 | <LOD | 6 | <LOD | 3 | <LOD | 73 | 95 | 19 | <LOD | 98 | 87 | 26 | <LOD | 174 | 443 | 23 |
| Eye shadow | China | <LOD | 6 | <LOD | 5 | <LOD | 2 | <LOD | 65 | <LOD | 50 | <LOD | 117 | <LOD | 54 | <LOD | 145 | 129 | 21 |
| Eye shadow | China | <LOD | 10 | <LOD | 9 | <LOD | 4 | <LOD | 109 | <LOD | 152 | <LOD | 920 | <LOD | 218 | <LOD | 252 | 424 | 34 |
| Lip or mouth products | USA | <LOD | 6 | <LOD | 5 | <LOD | 2 | <LOD | 62 | <LOD | 44 | <LOD | 33 | <LOD | 43 | <LOD | 137 | 151 | 20 |
| Lip or mouth products | USA | <LOD | 5 | <LOD | 4 | <LOD | 2 | <LOD | 60 | <LOD | 42 | 30 | 10 | <LOD | 40 | <LOD | 132 | 138 | 19 |
| Lip or mouth products | China | 13 | 3 | <LOD | 29 | <LOD | 5 | <LOD | 92 | <LOD | 142 | <LOD | 170 | <LOD | 163 | <LOD | 219 | 198 | 29 |
| Lip or mouth products | USA | <LOD | 6 | <LOD | 5 | 3 | 1 | <LOD | 73 | <LOD | 54 | <LOD | 31 | 232 | 56 | <LOD | 171 | 351 | 24 |
| Lip or mouth products | USA | <LOD | 8 | <LOD | 5 | <LOD | 3 | <LOD | 71 | <LOD | 95 | <LOD | 532 | <LOD | 119 | <LOD | 167 | 284 | 22 |
| Lip or mouth products | USA | <LOD | 5 | <LOD | 5 | <LOD | 2 | <LOD | 61 | <LOD | 43 | <LOD | 86 | <LOD | 50 | <LOD | 139 | 306 | 19 |
| Lip or mouth products | China | <LOD | 7 | <LOD | 5 | <LOD | 3 | <LOD | 81 | <LOD | 58 | <LOD | 36 | <LOD | 56 | <LOD | 180 | 223 | 26 |
| *Sample number (N=74): body paint (N=57); eye shadow (N=10); lip or mouth products (N=7) | | | | | | | | | | | | | | | | | | | |

Table S2. EMSL elemental concentrations in select cosmetic products detected by Inductively Coupled Plasma Mass Spectrometry (mg/kg)

| **Product** | **EMSL Results** | | | | | | | |
| --- | --- | --- | --- | --- | --- | --- | --- | --- |
|  | Arsenic (As) | Cadmium (Cd) | Lead  (Pb) | Nickel (Ni) | Chromium (Cr) | Antimony (Sb) | Cobalt (Co) | Silver (Ag) |
| Eye Shadow Pink | <LOQ | <LOQ | 5.68 mg/kg | 2.52 mg/kg | <LOQ | <LOQ | 0.86 mg/kg | <LOQ |
| Eye Shadow Orange | <LOQ | <LOQ | 5.42 mg/kg | 2.52 mg/kg | <LOQ | <LOQ | <LOQ | <LOQ |
| Eye Shadow Orange duplicate | <LOQ | <LOQ | 6.29 mg/kg | 3.67 mg/kg | <LOQ | <LOQ | 1.49 mg/kg | <LOQ |
| Eye Shadow Green | <LOQ | <LOQ | 5.80 mg/kg | 3.67 mg/kg | <LOQ | <LOQ | 1.44 mg/kg | <LOQ |
| Eye Shadow Yellow | <LOQ | <LOQ | 4.01 mg/kg | 1.32 mg/kg | <LOQ | <LOQ | <LOQ | <LOQ |
| Body Paint Glow-in-the-dark | 8.32 mg/kg | <LOQ | <LOQ | <LOQ | <LOQ | <LOQ | <LOQ | <LOQ |
| Body Paint Red glitter | <LOQ | <LOQ | <LOQ | <LOQ | <LOQ | 6.75 mg/kg | <LOQ | <LOQ |
| Body Paint Gold | <LOQ | <LOQ | 5.63 mg/kg | 5.79 mg/kg | <LOQ | <LOQ | 1.13 mg/kg | <LOQ |
| Body Paint Silver | <LOQ | <LOQ | 5.52 mg/kg | 1.74 mg/kg | <LOQ | <LOQ | 0.99 mg/kg | <LOQ |
| Body Paint Bronze | <LOQ | <LOQ | 5.08 mg/kg | 5.88 mg/kg | <LOQ | <LOQ | 1.83 mg/kg | <LOQ |
| * - Presence of chromium oxide in the sample prevents the inductively coupled plasma mass spectrometry analysis from determining the amount of lead in the sample because of the metal complexes that are formed in the solution | | | | | | | | |
| ** - Internal standard used during inductively coupled plasma mass spectrometry does not pass the QC because of the high dilution factor required by the amount of chromium in the sample | | | | | | | | |
| *** - Analyzed by X-ray fluorescence analysis | | | | | | | | |

Table S3. Elemental concentrations in select cosmetic products detected by Inductively Coupled Plasma Mass Spectrometry, analyzed at Brooks Applied Laboratory, Bothell, WA, USA (mg/kg).

|  | | | | | | | | | | | | | |
| --- | --- | --- | --- | --- | --- | --- | --- | --- | --- | --- | --- | --- | --- |
| Element | Method | Eye Shadow Pink | Eye Shadow Orange | Eye Shadow Orange duplicate | Eye Shadow Green | Eye Shadow Yellow | Body Paint Glow-in-the-dark | Body Paint Red glitter | Body Paint Gold | Body Paint Silver | Body Paint Bronze | Mean all products (mg/kg) | Detection frequency all products (%) |
| Antimony | Quantitative | 0.49 | 0.57 | 0.18 | 0.32 | 0.13 | 0.12 | 6.2 | 0.22 | 0.62 | 0.13 | 0.90 | 100% |
| Arsenic | Quantitative | 0.20^E^ | 0.25^E^ | 0.38^E^ | 0.42^E^ | 0.25^E^ | *0.08* | *0.079* | 0.48^E^ | 0.526 | 0.11^E^ | 0.28 | 10% |
| Cadmium | Quantitative | *0.027* | *0.025* | *0.027* | 0.034 | *0.026* | *0.026* | *0.026* | *0.028* | *0.028* | *0.027* | 0.027 | 0% |
| Mercury | Quantitative | 0.0019 | 0.00068 | 0.0013 | 0.00093 | *0.00016* | 0.0020 | *0.00015* | *0.00016* | *0.00016* | 0.00039E | 0.00079 | 50% |
| Methylmercury | Quantitative | *0.001* | *0.0009* | *0.001* | *0.001* | *0.0009* | *0.0011* | 0.0016^E^ | *0.0011* | *0.001* | *0.001* | 0.0011 | 0% |
| Nickel | Quantitative | 2.5 | 2.5 | 2.0 | 2.4 | 1.3 | *0.21* | *0.20* | 6.1 | 1.9 | 6.3 | 2.5 | 80% |
| Lead | Quantitative | 7.3 | 7.3 | 9.3 | 8.4 | 4.8 | 0.42^E^ | *0.15* | 5.7 | 6.0 | 5.4 | 5.5 | 80% |
| Antimony | Semi-quantitative | *0.5* | *0.5* | *0.5* | *0.5* | *0.5* | *0.5* | 6.3 | *0.5* | 0.72 | *0.5* | 1.1 | 20% |
| *Arsenic* | Semi-quantitative | *0.5* | *0.5* | *0.5* | *0.5* | *0.5* | *0.5* | *0.5* | *0.5* | *0.5* | *0.5* | *0.5* | 0% |
| *Cadmium* | Semi-quantitative | *0.5* | *0.5* | *0.5* | *0.5* | *0.5* | *0.5* | *0.5* | *0.5* | *0.5* | *0.5* | *0.5* | 0% |
| *Mercury* | Semi-quantitative | *0.5* | *0.5* | *0.5* | *0.5* | *0.5* | *0.5* | *0.5* | *0.5* | *0.5* | *0.5* | *0.5* | 0% |
| Nickel | Semi-quantitative | 3.0 | 2.7 | 2.5 | 2.9 | 1.8 | *0.5* | *0.5* | 5.6 | 2.2 | 6.6 | 2.8 | 80% |
| Lead | Semi-quantitative | 7.3 | 7.7 | 10.1 | 8.8 | 4.8 | *0.5* | *0.5* | 6.1 | 6.1 | 5.4 | 5.7 | 80% |
| Aluminum | Semi-quantitative | 1.9E+04 | 1.9E+04 | 3.0E+04 | 2.7E+04 | 1.7E+04 | 1.3E+03 | 1.4E+02 | 4.2E+04 | 4.3E+04 | 3.5E+04 | 2.3E+04 | 100% |
| Sodium | Semi-quantitative | 1.9E+03 | 3.7E+03 | 4.0E+03 | 1.9E+03 | 1.6E+03 | 1.4E+03 | 2.8E+02 | 2.4E+03 | 1.6E+03 | 2.1E+03 | 2.1E+03 | 100% |
| Barium | Semi-quantitative | 2.9E+02 | 1.3E+03 | 8.9E+02 | 1.4E+02 | 93 | 1.4 | *0.5* | 90 | 1.0E+02 | 72 | 294 | 90% |
| Calcium | Semi-quantitative | 5.1E+03 | 4.7E+03 | 1.1E+04 | 1.1E+04 | 5.6E+02 | 4.1E+02 | *1.3E+02* | 1.6E+02 | 1.4E+02 | 4.6E+02 | 3.4E+03 | 90% |
| Iron | Semi-quantitative | 5.0E+03 | 5.0E+03 | 5.7E+03 | 5.4E+03 | 3.3E+03 | 2.6E+02 | *5.0E+01* | 1.2E+04 | 6.0E+03 | 6.8E+04 | 1.1E+04 | 90% |

| Element | Method | Eye Shadow Pink | Eye Shadow Orange | Eye Shadow Orange duplicate | | Eye Shadow Green | Eye Shadow Yellow | Body Paint Glow-in-the-dark | Body Paint Red glitter | Body Paint Gold | | Body Paint Silver | Body Paint Bronze | | Mean all products (mg/kg) | Detection frequency all products (%) | |  |  |  |  |  |
| --- | --- | --- | --- | --- | --- | --- | --- | --- | --- | --- | --- | --- | --- | --- | --- | --- | --- | --- | --- | --- | --- | --- |
| Magnesium | Semi-quantitative | 3.5E+04 | 2.5E+04 | 2.4E+04 | | 2.4E+04 | 3.3E+04 | 2.0E+03 | *5.0E+01* | 9.2E+03 | | 1.3E+04 | 1.8E+04 | | 1.8E+04 | 90% | |  |  |  |  |  |
| Manganese | Semi-quantitative | 53 | 51 | 85 | | 84 | 45 | 4.2 | *0.5* | 75 | | 34 | 97 | | 53 | 90% | |  |  |  |  |  |
| Potassium | Semi-quantitative | 2.3E+04 | 2.8E+04 | 2.7E+04 | | 2.3E+04 | 2.2E+04 | 1.7E+02 | *5.0E+01* | 1.5E+04 | | 1.7E+04 | 1.3E+04 | | 1.7E+04 | 90% | |  |  |  |  |  |
| Rubidium | Semi-quantitative | 1730 | 2290 | 1780 | | 1740 | 2470 | 9.1 | *0.5* | 1620 | | 1720 | 1520 | | 1488 | 90% | |  |  |  |  |  |
| Strontium | Semi-quantitative | 15 | 25 | 23 | | 13 | 2.8 | 46 | *0.5* | 3.1 | | 2.9 | 3.3 | | 13 | 90% | |  |  |  |  |  |
| Titanium | Semi-quantitative | 5.5E+02 | 5.6E+02 | 4.9E+02 | | 4.6E+02 | 2.9E+02 | 8.5E+01 | *5.0E+00* | 4.9E+04 | | 4.6E+04 | 3.3E+04 | | 1.3E+04 | 90% | |  |  |  |  |  |
| Zinc | Semi-quantitative | 41 | 1230 | 22 | | 23 | 24 | 142000 | *13* | 27 | | 21 | 23 | | 14342 | 90% | |  |  |  |  |  |
| Zirconium | Semi-quantitative | 13 | 10 | 8.9 | | 9.7 | 1.4 | 2.3 | *1.3* | 6.5 | | 14 | 2.6 | | 7.0 | 90% | |  |  |  |  |  |
| Beryllium | Semi-quantitative | 3.6 | 3.3 | 6.7 | | 3.1 | 8.6 | 0.85 | *0.5* | 3.2 | | *0.5* | 3.5 | | 3.4 | 80% | |  |  |  |  |  |
| Cesium | Semi-quantitative | 14 | 18 | 16 | | 14 | 21 | *0.5* | *0.5* | 15 | | 15 | 15 | | 13 | 80% | |  |  |  |  |  |
| Cobalt | Semi-quantitative | 1.1 | 0.95 | 1.1 | | 1.1 | 0.52 | *0.5* | *0.5* | 1.3 | | 1.2 | 2.0 | | 1.0 | 80% | |  |  |  |  |  |
| Copper | Semi-quantitative | 0.87 | 1.4 | 2.3 | | 3.3 | *0.5* | 22 | *0.5* | 2.8 | | 2.4 | 2.0 | | 3.8 | 80% | |  |  |  |  |  |
| Niobium | Semi-quantitative | 18 | 22 | 24 | | 21 | 25 | *0.5* | *0.5* | 50 | | 54 | 17 | | 23 | 80% | |  |  |  |  |  |
| Silicon | Semi-quantitative | 3.8E+05 | 3.6E+05 | 3.1E+05 | | 2.9E+05 | 3.3E+05 | *5.0E+04* | *5.0E+04* | 9.0E+04 | | 1.1E+05 | 1.1E+05 | | 2.1E+05 | 80% | |  |  |  |  |  |
| Tantalum | Semi-quantitative | 2.1 | 3.2 | 3.4 | | 3.1 | 5.1 | *0.5* | *0.5* | 4.6 | | 4.8 | 3.1 | | 3.0 | 80% | |  |  |  |  |  |
| Thallium | Semi-quantitative | 0.61 | 0.93 | 0.90 | | 0.73 | 1.1 | *0.5* | *0.5* | 0.62 | | 0.72 | 0.67 | | 0.73 | 80% | |  |  |  |  |  |
| Tin | Semi-quantitative | 22 | 30 | 31 | | 27 | 38 | *0.5* | *0.5* | 28 | | 64 | 29 | | 27 | 80% | |  |  |  |  |  |
| Tungsten | Semi-quantitative | 22 | 30 | 34 | | 30 | 26 | *0.5* | *0.5* | 18 | | 21 | 16 | | 20 | 80% | |  |  |  |  |  |
| Cerium | Semi-quantitative | 7.5 | 7.7 | 3.0 | | 4.2 | *0.5* | 1.3 | *0.5* | 1.1 | | *0.5* | *0.5* | | 2.7 | 60% | |  |  |  |  |  |
| Element | Method | Eye Shadow Pink | Eye Shadow Orange | Eye Shadow Orange duplicate | | Eye Shadow Green | Eye Shadow Yellow | Body Paint Glow-in-the-dark | Body Paint Red glitter | Body Paint Gold | | Body Paint Silver | Body Paint Bronze | | Mean all products (mg/kg) | Detection frequency all products (%) | |  |  |  |  |  |
| Thorium | Semi-quantitative | 1.9 | 2.5 | 1.3 | | 2.2 | *0.5* | 0.61 | *0.5* | *0.5* | | *0.5* | *0.5* | | 1.1 | 50% | |  |  |  |  |  |
| Vanadium | Semi-quantitative | 19 | 19 | 15 | | 15 | 13 | *13* | *13* | *13* | | *13* | *13* | | 14 | 50% | |  |  |  |  |  |
| Hafnium | Semi-quantitative | 0.55 | 0.59 | *0.5* | | 0.67 | *0.5* | *0.5* | *0.5* | *0.5* | | 0.95 | *0.5* | | 0.58 | 40% | |  |  |  |  |  |
| Neodymium | Semi-quantitative | 2.8 | 3.0 | 0.83 | | 1.4 | *0.5* | *0.5* | *0.5* | *0.5* | | *0.5* | *0.5* | | 1.1 | 40% | |  |  |  |  |  |
| Uranium | Semi-quantitative | 0.67 | 0.71 | 1.2 | | 1.3 | *0.5* | *0.5* | *0.5* | *0.5* | | *0.5* | *0.5* | | 0.69 | 40% | |  |  |  |  |  |
| Bismuth | Semi-quantitative | *0.5* | *0.5* | 0.55 | | 0.59 | *0.5* | *0.5* | *0.5* | *0.5* | | *0.5* | *0.5* | | 0.51 | 20% | |  |  |  |  |  |
| Praseodimium | Semi-quantitative | 0.85 | 0.84 | *0.5* | | *0.5* | *0.5* | *0.5* | *0.5* | *0.5* | | *0.5* | *0.5* | | 0.57 | 20% | |  |  |  |  |  |
| Samarium | Semi-quantitative | 0.6 | 0.52 | *0.5* | | *0.5* | *0.5* | *0.5* | *0.5* | *0.5* | | *0.5* | *0.5* | | 0.51 | 20% | |  |  |  |  |  |
| Molybdenum | Semi-quantitative | *0.5* | *0.5* | *0.5* | | *0.5* | *0.5* | *0.5* | *0.5* | 0.68 | | *0.5* | *0.5* | | 0.52 | 10% | |  |  |  |  |  |
| Phosphorus | Semi-quantitative | *5.0E+02* | *5.0E+02* | *5.0E+02* | | *5.0E+02* | *5.0E+02* | *5.0E+02* | *5.0E+02* | *5.0E+02* | | 5.0E+02 | *5.0E+02* | | 5.0E+02 | 10% | |  |  |  |  |  |
| Sulfur | Semi-quantitative | *1.3E+04* | *1.3E+04* | *1.3E+04* | | *1.3E+04* | *1.3E+04* | 7.2E+04 | *1.3E+04* | *1.3E+04* | | *1.3E+04* | *1.3E+04* | | 1.9E+04 | 10% | |  |  |  |  |  |
| *Boron* | Semi-quantitative | *50* | *50* | *50* | | *50* | *50* | *50* | *50* | *50* | | *50* | *50* | | *50* | 0% | |  |  |  |  |  |
| *Chromium* | Semi-quantitative | *13* | *13* | *13* | | *13* | *13* | *13* | *13* | *13* | | *13* | *13* | | *13* | 0% | |  |  |  |  |  |
| *Dysprosium* | Semi-quantitative | *0.5* | *0.5* | *0.5* | | *0.5* | *0.5* | *0.5* | *0.5* | *0.5* | | *0.5* | *0.5* | | *0.5* | 0% | |  |  |  |  |  |
| *Erbium* | Semi-quantitative | *0.5* | *0.5* | *0.5* | | *0.5* | *0.5* | *0.5* | *0.5* | *0.5* | | *0.5* | *0.5* | | *0.5* | 0% | |  |  |  |  |  |
| *Europium* | Semi-quantitative | *0.5* | *0.5* | *0.5* | | *0.5* | *0.5* | *0.5* | *0.5* | *0.5* | | *0.5* | *0.5* | | *0.5* | 0% | |  |  |  |  |  |
| *Gadolinium* | Semi-quantitative | *0.5* | *0.5* | *0.5* | | *0.5* | *0.5* | *0.5* | *0.5* | *0.5* | | *0.5* | *0.5* | | *0.5* | 0% | |  |  |  |  |  |
| *Gold* | Semi-quantitative | *0.5* | *0.5* | *0.5* | | *0.5* | *0.5* | *0.5* | *0.5* | *0.5* | | *0.5* | *0.5* | | *0.5* | 0% | |  |  |  |  |  |
| *Holmium* | Semi-quantitative | *0.5* | *0.5* | *0.5* | | *0.5* | *0.5* | *0.5* | *0.5* | *0.5* | | *0.5* | *0.5* | | *0.5* | 0% | |  |  |  |  |  |
| Element | Method | Eye Shadow Pink | Eye Shadow Orange | Eye Shadow Orange duplicate | | Eye Shadow Green | Eye Shadow Yellow | Body Paint Glow-in-the-dark | Body Paint Red glitter | Body Paint Gold | | Body Paint Silver | Body Paint Bronze | | Mean all products (mg/kg) | Detection frequency all products (%) | |  |  |  |  |  |
| *Lutetium* | Semi-quantitative | *0.5* | *0.5* | *0.5* | | *0.5* | *0.5* | *0.5* | *0.5* | *0.5* | | *0.5* | *0.5* | | *0.5* | 0% | |  |  |  |  |  |
| *Osmium* | Semi-quantitative | *0.5* | *0.5* | *0.5* | | *0.5* | *0.5* | *0.5* | *0.5* | *0.5* | | *0.5* | *0.5* | | *0.5* | 0% | |  |  |  |  |  |
| *Palladium* | Semi-quantitative | *0.5* | *0.5* | *0.5* | | *0.5* | *0.5* | *0.5* | *0.5* | *0.5* | | *0.5* | *0.5* | | *0.5* | 0% | |  |  |  |  |  |
| *Platinum* | Semi-quantitative | *0.5* | *0.5* | *0.5* | | *0.5* | *0.5* | *0.5* | *0.5* | *0.5* | | *0.5* | *0.5* | | *0.5* | 0% | |  |  |  |  |  |
| *Rhenium* | Semi-quantitative | *0.5* | *0.5* | *0.5* | | *0.5* | *0.5* | *0.5* | *0.5* | *0.5* | | *0.5* | *0.5* | | *0.5* | 0% | |  |  |  |  |  |
| *Ruthenium* | Semi-quantitative | *0.5* | *0.5* | *0.5* | | *0.5* | *0.5* | *0.5* | *0.5* | *0.5* | | *0.5* | *0.5* | | *0.5* | 0% | |  |  |  |  |  |
| *Selenium* | Semi-quantitative | *5* | *5* | *5* | | *5* | *5* | *5* | *5* | *5* | | *5* | *5* | | *5* | 0% | |  |  |  |  |  |
| *Silver* | Semi-quantitative | *0.5* | *0.5* | *0.5* | | *0.5* | *0.5* | *0.5* | *0.5* | *0.5* | | *0.5* | *0.5* | | *0.5* | 0% | |  |  |  |  |  |
| *Terbium* | Semi-quantitative | *0.5* | *0.5* | *0.5* | | *0.5* | *0.5* | *0.5* | *0.5* | *0.5* | | *0.5* | *0.5* | | *0.5* | 0% | |  |  |  |  |  |
| *Thulium* | Semi-quantitative | *0.5* | *0.5* | *0.5* | | *0.5* | *0.5* | *0.5* | *0.5* | *0.5* | | *0.5* | *0.5* | | *0.5* | 0% | |  |  |  |  |  |
| *Ytterbium* | Semi-quantitative | *0.5* | *0.5* | *0.5* | | *0.5* | *0.5* | *0.5* | *0.5* | *0.5* | | *0.5* | *0.5* | | *0.5* | 0% | |  |  |  |  |  |
| Values with an "E" indicate the analyte was detected by the instrument. Result is > the Method Detecting Limit but ≤ Method Reporting Limit. Result reported but considered an estimate |  |  |  |  | |  |  |  |  |  | |  |  | |  |  | |  |  |  |  |  |
| Values in *italics* and grey shade indicate the result is ≤ Method Detecting Limit (MDL). Result reported as the MDL. | | | | | | | | | | |  | | | | | | |  |  |  |  |  |
|  | | | | |  | |  | |  |  | | | |  | | |  |  |  |  |  |  |

Table S4. Relative percent deviation as a measure of intra-laboratory comparison of inductively coupled plasma mass spectrometry elemental analysis of select costume cosmetics.

| Element | Eye Shadow Pink | Eye Shadow Orange | Eye Shadow Orange duplicate | Eye Shadow Green | Eye Shadow Yellow | Body Paint Glow-in-the-dark | Body Paint Red glitter | Body Paint Gold | Body Paint Silver | Body Paint Bronze |
| --- | --- | --- | --- | --- | --- | --- | --- | --- | --- | --- |
| Arsenic | -- | -- | -- | -- | -- | -- | -- | -- | -- | -- |
| Cadmium | -- | -- | -- | -- | -- | -- | -- | -- | -- | -- |
| Lead | 17.2 | 21.3 | 27.6 | 25.7 | 12.5 | -- | -- | 0.4 | 6.4 | 4.6 |
| Nickel | 1.7 | 0.0 | 41.7 | 28.7 | 1.1 | -- | -- | 3.2 | 7.7 | 4.7 |
| Chromium | -- | -- | -- | -- | -- | -- | -- | -- | -- | -- |
| Antimony | -- | -- | -- | -- | -- | -- | 6.0 | -- | -- | -- |
| Cobalt | 14.7 | 12.1 | 20.7 | 20.2 | 30.0 | -- | -- | 7.7 | 11.8 | 5.9 |
| Silver | -- | -- | -- | -- | -- | -- | -- | -- | -- | -- |
| "--" = Non-detect |  |  |  |  |  |  |  |  |  |  |

Table S5. Physico-chemical properties required for SkinPerm modeling

|  | | | | | | |  |  |  |  |  |  |  |
| --- | --- | --- | --- | --- | --- | --- | --- | --- | --- | --- | --- | --- | --- |
| **Element** | **Substance** | **Chemical Formula** | | **CAS #** | | **Molecular Weight (g/mol)** | **Temperature (C)** | **VP (Pa)** | **Water Solubility (mg/L)** | **LogKow at  skin pH 5.5** | **Density  (mg/cm^3^)** | **Permeability Coefficient  (cm/hr)** | **References** |
| Pb | Lead acetate^a^ | | Pb(C_2_H_3_O_2_)_2_ | 301-04-2 | | 325.28 | 25 | 9.60E-02 | 4.43E+05 | -0.083 | 3250 | 4.20E-06 | [4, 8, 21, 30] |
| Hg | Mercury (II) chloride^b^ | | HgCl_2_ | 7487-94-7 | | 271.52 | 25 | 1.33E+02 | 7.31E+04 | 0.22 | 5600 | 9.30E-04 | [5, 21, 27, 30] |
| As | Arsenic trioxide^c^ | | As_2_O_3_ | 1327-53-3 | | 197.841 | 25 | 3.29E-02 | 1.70E+04 | -0.13 | 5778 | 1.00E-03 | [1, 9, 17, 23, 30] |
| Cd | Cadmium sulfate^d^ | | CdSO_4_ | 10124-36-4 | | 208.47 | 25 | 1.33E+03 | 7.67E+05 | 0.21 | 4690 | 1.10E-03 | [2, 20, 24, 30] |
| Ni | Nickel (II) chloride^e^ | | NiCl_2_ | 7718-54-9 | | 129.6 | 25 | 1.33E+02 | 6.75E+05 | 0.05 | 3510 | 1.00E-03 | [6, 13, 21, 30] |
| Co | Cobalt (II) chloride | | CoCl_2_ | 7646-79-9 | | 129.84 | 25 | 1.00E+04 | 5.62E+05 | 0.85 | 3360 | 4.00E-04 | [3, 12, 21, 26, 30] |
| Cr(III) | Chromium III oxide^f^ | | Cr_2_O_3_ | 1308-38-9 | | 151.99 ^6^ | 25 | 5.80E-10 | 5.80E+05 | 1.16 | 5220 | 2.52E-07 | [10, 18, 25, 30, 31] |
| Cr(VI) | Potassium chromate^g^ | | K_2_CrO_4_ | 7789-00-6 | | 194.19 | 25 | 0.00E+00 | 6.50E+05 | 2.23 | 2730 | 1.00E-03 | [11, 19, 28, 30] |
| Sb | Antimony trioxide^h^ | | Sb_2_O_3_ | 1309-64-4 | | 291.52 | 25 | 1.33E+02 | 2.21E+01 | 0 | 5200 | 2.92E-03 | [15, 16, 22, 29, 32] |
| Ag | Silver chloride^i^ | | AgCl | 7783-90-6 | | 143.34 | 25 | 6.32E-16 | 1.90E+00 | 0.54 | 5560 | 3.50E-04 | [7, 14, 21, 30] |
| ^a^ vapor pressure at 520 ^o^C | | |  |  | |  |  |  |  |  |  |  |  |
| ^b^ vapor pressure at 136.2 ^o^C | | |  |  | |  |  |  |  |  |  |  |  |
| ^c^ water solubility at 16 ^o^C according to Agency for Toxic Substances and Disease Registry | | | | |  |  |  |  |  |  |  |  |  |
| ^d^ Vapor pressure, Log Kow and permeability coefficient for CdCl_2_; log Kow at 20 ^o^C | | | | | | |  |  |  |  |  |  |  |
| ^e^ vapor pressure at 671^o^C | | |  |  | |  |  |  |  |  |  |  |  |
| ^f^ water solubility as chromium perchlorate; logkow as chromium chloride | | | | | |  |  |  |  |  |  |  |  |
| ^g^ logkow as chromium (VI) trioxide; permeability coefficient as Chromium VI element | | | | | | |  |  |  |  |  |  |  |
| ^h^ vapor pressure and logKow for elemental antimony; used permeability coefficient for arsenic; water solubility based on antimony (III) chloride | | | | | | | | | |  |  |  |  |
| ^i^ Density @ 20^o^C; Permeability coefficient for silver nitrate | | | |  | |  |  |  |  |  |  |  |  |

**References** *[please include all references relevant to the data described here; references are not limited]*

1. ATSDR (Agency for Toxic Substances and Disease Registry), Toxicological Profile for Lead. U.S. Department of Health and Human Services, Public Health Service, Agency for Toxic Substances and Disease Registry, Atlanta, GA, 2007b, pp. 1-582.
2. ATSDR (Agency for Toxic Substances and Disease Registry), Toxicological Profile for Mercury. U.S. Department of Health and Human Services, Public Health Service, Agency for Toxic Substances and Disease Registry, Atlanta, GA, 1999, pp. 1-676.
3. ATSDR (Agency for Toxic Substances and Disease Registry), Toxicological Profile for Arsenic. U.S. Department of Health and Human Services, Public Health Service, Agency for Toxic Substances and Disease Registry, Atlanta, GA, 2007, pp. 1-559.
4. ATSDR (Agency for Toxic Substances and Disease Registry), Toxicological Profile for Cadmium. U.S. Department of Health and Human Services, Public Health Service, Agency for Toxic Substances and Disease Registry, Atlanta, GA, 2012, pp. 1-487.
5. ATSDR (Agency for Toxic Substances and Disease Registry), Toxicological Profile for Nickel. U.S. Department of Health and Human Services, Public Health Service, Agency for Toxic Substances and Disease Registry, Atlanta, GA, 2005, pp. 1-397.
6. ATSDR (Agency for Toxic Substances and Disease Registry), Toxicological Profile for Cobalt. U.S. Department of Health and Human Services, Public Health Service, Agency for Toxic Substances and Disease Registry, Atlanta, GA, 2004, pp. 1-486.
7. ATSDR (Agency for Toxic Substances and Disease Registry), Toxicological Profile for Silver. U.S. Department of Health and Human Services, Public Health Service, Agency for Toxic Substances and Disease Registry, Atlanta, GA, 2012, pp. 1-157.
8. Guy. R.H., Hotynek, J.J., Hinz, R.S., and Lorence, C.R. 1999. Metals and the Skin: Topical Effects and Systemic Absorption. CRC Press
9. Hayes, A. Wallace, and Kruger, C.L. eds. 2014.  Hayes' principles and methods of toxicology. CRC Press, 2014.
10. Lide, D. R. (2007). CRC handbook of chemistry and physics: 88th Edition. Boca Raton, Fla: CRC Press.
11. NTP (National Toxicology Program). 2005. Antimony Trioxide [CAS No. 1309-64-4] Brief Review of Toxicological Literature.
12. Perez, A., Nembhard, M., Monnot, A., Bator, D., Madonick, E., and Gaffney, S.H. (in press). Child and adult exposure and health risk evaluation following the use of metal- and metalloid-containing costume cosmetics sold in the United States. Reg. Toxicol. Pharmacol. *In press*
13. MAK Value Documentation. 2014. Chromium(III) and its Inorganic Compounds. The MAK Collection for Occupational Health and Safety. 1–38.
14. ChemSpider, unknown. Silver Chloride. ChemSpider ID: 22967. Accessed September 14, 2016: <http://www.chemspider.com/Chemical-Structure.22967.html?rid=c1905d5b-58ef-4e62-a969-77c0eae31131>.
15. ChemSpider, unknown. Arsenic Trioxide. ChemSpider ID: 229103. Accessed September 14, 2016: <http://www.chemspider.com/Chemical-Structure.229103.html?rid=1e55e09e-5b6a-4a4e-8f99-5a1441d8b759&page_num=0>.
16. ChemSpider, unknown. Chromium Trioxide. ChemSpider ID: 14212. Accessed September 14, 2016: <http://www.chemspider.com/Chemical-Structure.14212.html?rid=e6a7aa15-3fae-4e74-a77d-53eb2672906e>.
17. ChemSpider, unknown. Chromium Chloride. ChemSpider ID: 23193. Accessed September 14, 2016: <http://www.chemspider.com/Chemical-Structure.23193.html?rid=f97603a4-a136-4678-91ba-d3f9f23025fc&page_num=0>.
18. ChemSpider, unknown. Cobalt(II) Chloride. ChemSpider ID: 22708. Accessed September 14, 2016: <http://www.chemspider.com/Chemical-Structure.22708.html?rid=798f2db3-3a19-4e07-8a7b-466b56b29645>.
19. ChemSpider, unknown. Nickel(II) Chloride. ChemSpider ID: 22796. Accessed September 14, 2016: <http://www.chemspider.com/Chemical-Structure.22796.html?rid=3b9219a1-0756-4b6a-9a8e-cbc74fc631f6&page_num=0>.
20. ChemlDplus, unknown. Lead Acetate. RN: 301-04-2. Accessed September 14, 2016: <https://chem.nlm.nih.gov/chemidplus/rn/301-04-2>.
21. HSDB (Hazardous Substance Data Bank), 2005. Mercuric Chloride. CASRN: 7487-94-7. Octanol/Water Partition Coefficient. Accessed September 14, 2016: <http://toxnet.nlm.nih.gov/cgi-bin/sis/search2/r?dbs+hsdb:@term+@DOCNO+33>.
22. HSDB (Hazardous Substance Data Bank), 2003. Arsenic Trioxide. CASRN: 1327-53-3. Vapor Pressure. Accessed September 14, 2016: <http://toxnet.nlm.nih.gov/cgi-bin/sis/search2/r?dbs+hsdb:@term+@DOCNO+419>.
23. HSDB (Hazardous Substance Data Bank), 2012. Cadmium Chloride. CASRN: 10108-64-2. Octanol/Water Partition Coefficient. Accessed September 14, 2016: <http://toxnet.nlm.nih.gov/cgi-bin/sis/search2/r?dbs+hsdb:@term+@DOCNO+278>.
24. GSI, 2011. Arsenic. CAS No. 7440382. Accessed September 14, 2016: <http://www.gsi-net.com/en/publications/gsi-chemical-database/single/34-arsenic.html>.
25. GSI, 2016. Chromium (VI). CAS No. 18540299. Accessed September 14, 2016: <http://www.gsi-net.com/en/publications/gsi-chemical-database/single/136-chromium-vi.html>.
26. GSI, 2015. Chromium (III) (Total Chromium). CAS No. 7440473. Accessed September 14, 2016: <http://www.gsi-net.com/en/publications/gsi-chemical-database/single/135-chromium-iii-total-chromium.html>.
27. ILS (Integrated Laboratory Systems), 2005. Antimony Trioxide [CAS No. 1309-64-4]. Brief Review of Toxicological Literature. July 2005. National Toxicology Program, National Institute of Environmental Health Sciences, National Institutes of Health, U.S. Department of Health and Human Services, Research Triangle Park, NC.
28. ECHC (Environment Canada Health Canada), 2010. Screening Assessment for the Challenge: Antimony Trioxide (Antimony Oxide). Chemical Abstracts Service Registry Number: 1309-64-4. September 2010. Accessed September 14, 2016: <https://www.ec.gc.ca/ese-ees/9889ABB5-3396-435B-8428-F270074EA2A7/batch9_1309-64-4_en.pdf>.
29. HSDB (Hazardous Substance Data Bank), 2005. Potassium Chromate. CASRN: 7789-00-6. Vapor Pressure. Accessed September 14, 2016: <http://toxnet.nlm.nih.gov/cgi-bin/sis/search2/r?dbs+hsdb:@term+@DOCNO+1249>.
30. HSDB (Hazardous Substance Data Bank), 2004. Cobaltous Chloride. CASRN: 7646-79-9. Vapor Pressure. Accessed September 14, 2016: <http://toxnet.nlm.nih.gov/cgi-bin/sis/search2/r?dbs+hsdb:@term+@DOCNO+1000>.
31. HSDB, 2016. Chromium (III) Oxide. CASRN: 1308-38-9. Molecular Weight. Accessed September 14, 2016: <http://toxnet.nlm.nih.gov/cgi-bin/sis/search2/r?dbs+hsdb:@term+@DOCNO+1619>.
32. EBRC, 2007. Health Risk Assessment Guidance for Metals (HERAG). Fact Sheet: 01. Assessment of Occupational Dermal Exposure and Dermal Absorption for Metals and Inorganic Metal Compounds. August 2007. Accessed September 14, 2016: <http://www.ebrc.de/downloads/HERAG_FS_01_August_07.pdf>.
33. HSDB (Hazardous Substance Data Bank), 2013. Antimony Trioxide. CASRN: 1309-64-4. Chemical/Physical Properties. Accessed September 14, 2016: <https://toxnet.nlm.nih.gov/cgi-bin/sis/search2/r?dbs+hsdb:@term+@DOCNO+436>
